# Supplementary material for: Emptying Dirac valleys in bismuth using high magnetic fields
Source: Nat Commun. 2017 May 19;8:15297. doi: 10.1038/ncomms15297 (PMC5454462; doi:10.1038/ncomms15297)
Supplement: Supplementary Information — Supplementary Figures, Supplementary Table, Supplementary Notes and Supplementary References [file ncomms15297-s1.pdf]

SUPPLEMENTARY NOTE 1: THEORETICAL

The theoretical model used here is basically the same as that used in Supplementary Ref. 3. A minor difference is that we have replaced the scalar parameters V', which is the correction to the additional g-factor in the lowest Landau level, by a tensor:

$$\mathrm{V}' = \begin{pmatrix} V_1' & 0 & 0 \\ 0 & V_2' & V_4' \\ 0 & V_4' & V_3' \end{pmatrix}. \tag{1}$$

The values of parameters used in the present work are given in Supplementary table 1. Note also the band gap $2\Delta = 15.3$ meV, the electron-hole hybridization $E_0 = 38.5$ meV, and the interband coupling $V = 0.25$. (For details, see Supplementary Ref. 3,4.)

Supplementary Fig. 3(d) in the main text was calculated assuming an experimental misalignment. In order to find the best match with experiment, it was assumed that the rotation axis was not perfectly perpendicular to the magnetic field. The angle between them was taken to be $\theta = 92°$. Also, it was assumed that the trigonal axis of the sample was be $\theta = 2°$ off from the rotation axis in the direction of $\varphi = -10°$ from the original binary axis. These assumptions led to a map in excellent agreement with the experimental result.

Our model is essentially equivalent to the model introduced by Vecchi-Pereira-Dresselhaus (VPD)[5]. The model used in Ref.[1] has the same origin, with one important difference: the correction to the g-factor, which gives the quadratic field-dependence, was omitted. This has important consequences. The field dependence of the lowest Landau level of the three electron pockets is very different in the two models. The previous model leads to a simultaneous evacuation of all electron and hole landau levels at 88T for $B \parallel$ binary. By contrast, the present model indicates the correction to the g-factor, V', plays an important role at high fields, and leads to empty valleys at around 55T for $B \parallel$ binary and 40T for $B \parallel$ bisectrix.

SUPPLEMENTARY NOTE 2: TEMPERATURE-DEPENDENCE OF MAGNETORESISTANCE AS B ALONG BINARY AND BISECTRIX

Supplementary Fig. 1 shows the magnetoresistance along binary and bisectrix orientations for different temperatures of one of the two samples. As seen in the figure, the drop

Supplementary Table 1. **Values of parameters used in the present work**. The upper table is those for electrons and the lower one for holes. The m and M are all normalized by the bare mass of the electron. The g' and V' are zero dimensions.

i	1	2	3	4
m_i	0.00124	0.257	0.00585	-0.0277
g'_i	-7.26	24.0	-7.92	9.20
V'_i	-0.0688	-0.0438	-0.0625	0.00

i	1	3
M_i	0.0698	0.743
M_{si}	0.0319	10000

in magnetoresistance, which is the main result of this study persists up to a temperature significantly higher than what is required to wipe out quantum oscillations associated with the hole pocket. This implies that the drop in magnetoresistance is not simply due to the evacuation of a holme Landau level as previously suggested[1].

The absence of metal-to-semiconductor transition (Fig. 1 f in the main article) is in agreement with the expectations of the theoretical model used here. But, it is in contradiction with the expectations of the theoretical model used by Miura and Hiruma[1] in which, such a transition should occur at 88 T. Previously, far-infrared transmission measurement had detected a small anomaly at 88 T and this was interpreted as the signature of a metal-insulator transition[2].

SUPPLEMENTARY NOTE 3: DROP OF THE MAGNETORESISTANCE AS A CONSEQUENCE OF EMPTYING VALLEYS

In the semi-classical picture, one can qualitatively explain how emptying valleys can lead to a drop in magnetoresistance. The magneto-conductivity along the trigonal direction σ_{33} is given as[6]

$$\sigma_{33}^{\mathrm{bin}} = \frac{n_a e \mu_3}{1+(\mu_2\mu_3-\mu_4^2)B^2} + 2\frac{n_b e \mu_3}{1+(3\mu_1\mu_3+\mu_2\mu_3-\mu_4^2)B^2/4} + \frac{n_h e \nu_3}{1+\nu_1\nu_3 B^2}, \tag{2}$$

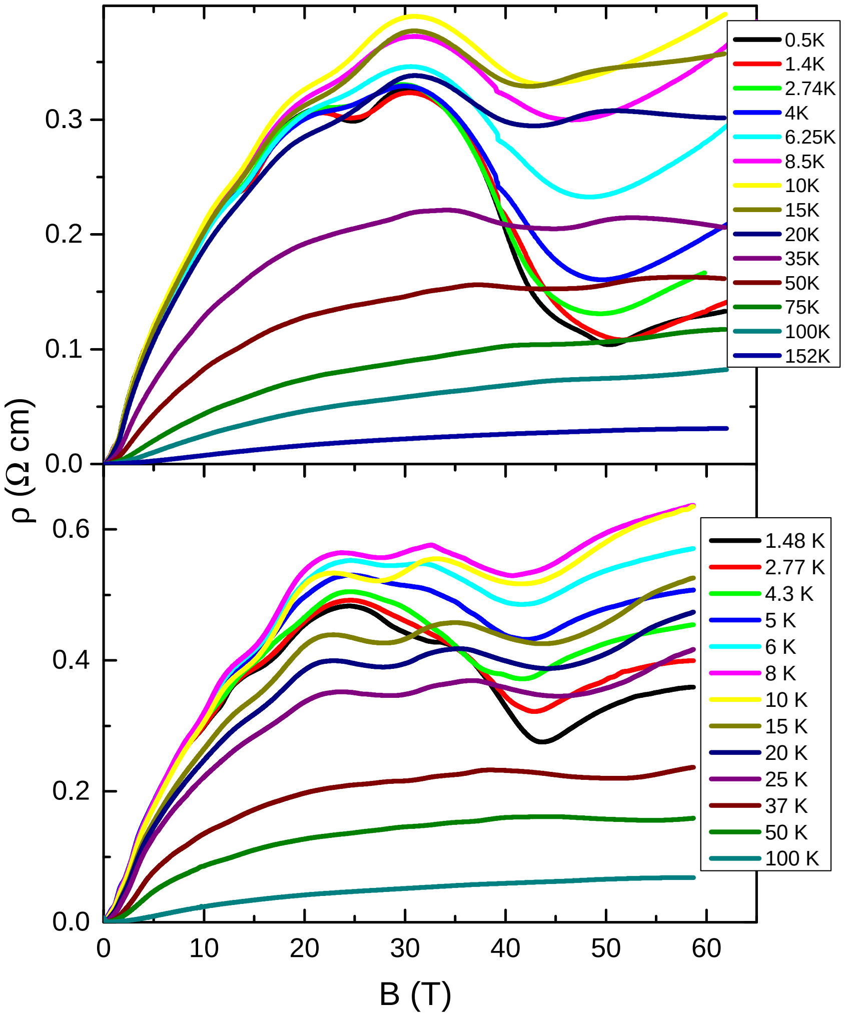

Supplementary Figure 1. **Temperature-dependence of magnetoresistance as B along binary and bisectrix** (a) Temperature-dependent Magnetoresistance for $B \parallel$ binary(sample #2) and (b)for $B \parallel$ bisectrix(sample#3) at different temperatures.

for $B \parallel$ binary axis, and :

$$\sigma_{33}^{\text{bis}} = \frac{n_a e \mu_3}{1 + \mu_1 \mu_3 B^2} + 2\frac{n_b e \mu_3}{1 + (\mu_1\mu_3 + 3\mu_2\mu_3 - 3\mu_4^2)B^2/4} + \frac{n_h e \nu_3}{1 + \nu_1 \nu_3 B^2}. \quad (3)$$

for $B \parallel$ bisectrix axis. At high fields ($\mu_i\mu_j B^2 \gg 1$), σ_{33} can be approximated as:

$$\sigma_{33}^{\rm bin} \simeq \frac{e}{B^2}\left[\left(\frac{1}{\mu_2}+\frac{1}{\nu_1}\right)n_a + 2\left(\frac{4}{3\mu_1+\mu_2}+\frac{1}{\nu_1}\right)n_b\right]$$
$$= \frac{en_h}{B^2}\left(\frac{f_a}{\mu_a}+2\frac{f_b}{\mu_b}\right), \quad (4)$$

, for $B \parallel$ binary axis, and:

$$\sigma_{33}^{\rm bis} \simeq \frac{e}{B^2}\left[\left(\frac{1}{\mu_1}+\frac{1}{\nu_1}\right)n_a + 2\left(\frac{4}{\mu_1+3\mu_2}+\frac{1}{\nu_1}\right)n_b\right]$$
$$= \frac{en_h}{B^2}\left(\frac{f_a}{\mu'_a}+2\frac{f_b}{\mu'_b}\right), \quad (5)$$

for $B \parallel$ bisectrix axis. It is assumed that $\mu_4 \ll \mu_{1,3}$. n_a, n_b, and n_c are the carrier densities of electron a, b, and c. (For $B \parallel$ binary and $B \parallel$ bisectrix, $n_b = n_c$.) The total number of electron carriers is equal to the number of hole carriers, i.e., $n_h = n_a + n_b + n_c$. The fraction of each electron carriers are:

$$f_a = \frac{n_a}{n_a+2n_b}, \quad f_b = \frac{n_b}{n_a+2n_b}. \quad (6)$$

We introduced the effective mobility for each electron pocket

$$\frac{1}{\mu_a} = \frac{1}{\mu_2}+\frac{1}{\nu_1}, \quad (7)$$
$$\frac{1}{\mu_b} = \frac{4}{3\mu_1+\mu_2}+\frac{1}{\nu_1}, \quad (8)$$
$$\frac{1}{\mu'_a} = \frac{1}{\mu_1}+\frac{1}{\nu_1}, \quad (9)$$
$$\frac{1}{\mu'_b} = \frac{4}{\mu_1+3\mu_2}+\frac{1}{\nu_1}. \quad (10)$$

Above the emptying field, $B > B_{\rm empt}$, the valley b and c are emptied for $B \parallel$ binary, while only the valley a is emptied for $B \parallel$ bisectrix. Therefore:

$$\sigma_{33}^{\rm bin} = \frac{en_h}{B^2}\frac{1}{\mu_a}, \quad (11)$$
$$\sigma_{33}^{\rm bis} = 2\frac{en_h}{B^2}\frac{1}{\mu'_b}. \quad (12)$$

The ratio of the magnetoresistivity above $B_{\rm empt}$ to that below $B_{\rm empt}$, $\Delta\rho = \rho_{33,\rm above}/\rho_{33,\rm below}$, is then given by

$$\Delta\rho_{33}^{\rm bin} \simeq \frac{\sigma_{33,\rm below}^{\rm bin}}{\sigma_{33,\rm above}^{\rm bin}} = \left(f_a + 2\frac{\mu_a}{\mu_b}f_b\right) \quad (13)$$
$$\Delta\rho_{33}^{\rm bis} \simeq \frac{\sigma_{33,\rm below}^{\rm bis}}{\sigma_{33,\rm above}^{\rm bis}} = \frac{1}{2}\left(\frac{\mu'_b}{\mu'_a}f_a + 2f_b\right). \quad (14)$$

The drop of resistivity, $\Delta\rho_{33} < 1$, occurs when

$$\mu_a < \mu_b \quad \text{for } B \parallel \text{binary}, \tag{15}$$

$$\mu'_a > \mu'_b \quad \text{for } B \parallel \text{bisectrix}, \tag{16}$$

since $f_a + 2f_b = 1$ by definition. If we write these conditions in terms of the original mobility tensors:

$$(15): \frac{1}{\mu_2} > \frac{4}{3\mu_1 + \mu_2} \Rightarrow \mu_1 > \mu_2 \tag{17}$$

$$(16): \frac{1}{\mu_1} < \frac{4}{\mu_1 + 3\mu_2} \Rightarrow \mu_2 < \mu_1. \tag{18}$$

In other words, the only necessary condition for the resistivity drop is $\mu_1 > \mu_2$. Even if we do not know the magnitude of μ_1 and μ_2 at high field, it is quite natural to expect $\mu_1 > \mu_2$ in bismuth, given the huge anisotropy at low field. Note that the factor 1/2 in Supplementary Eq. 14 suggests that the resistivity drop for $B \parallel$ binary is basically larger than that for $B \parallel$ bisectrix by a factor of 2, which agree with the experimental tendency.

The argument above shows that the transfer of carriers between valleys can qualitatively explain the drop in magnetoresistance. However, the actual situation is more complex. The total number of carriers n_h changes by the magnetic field, and a factor $\Delta n = n_{h,\text{below}}/n_{h,\text{above}}$ should be multiplied in Supplementary Eqs. (13) and (14). In the present case, $\Delta n > 1$ for both $B \parallel$ binary and $B \parallel$ bisectrix, so that it reduces the amplitude of the drop. The competition between these two factors, the anisotropy of the mobility and the field-dependent n_h, strongly depends on the field dependence of the mobility, about which we do not have enough information.

SUPPLEMENTARY NOTE 4: FIELD DEPENDENCE OF MOBILITY AND THE DROP OF MAGNETORESISTANCE IN THE NON-INTERACTING SEMICLASSICAL PICTURE

As discussed above, the drop of the magnetoresistance can be explained qualitatively as a result of the transfer of carriers between two valleys whose relevant components of mobility tensor are very different. But, is this enough for a quantitative explanation of the magnetoresistance? In this section, we will now examine how successful the semiclassical

picture is in quantitatively explaining the magnitude of the magnetoresistance and its field dependence.

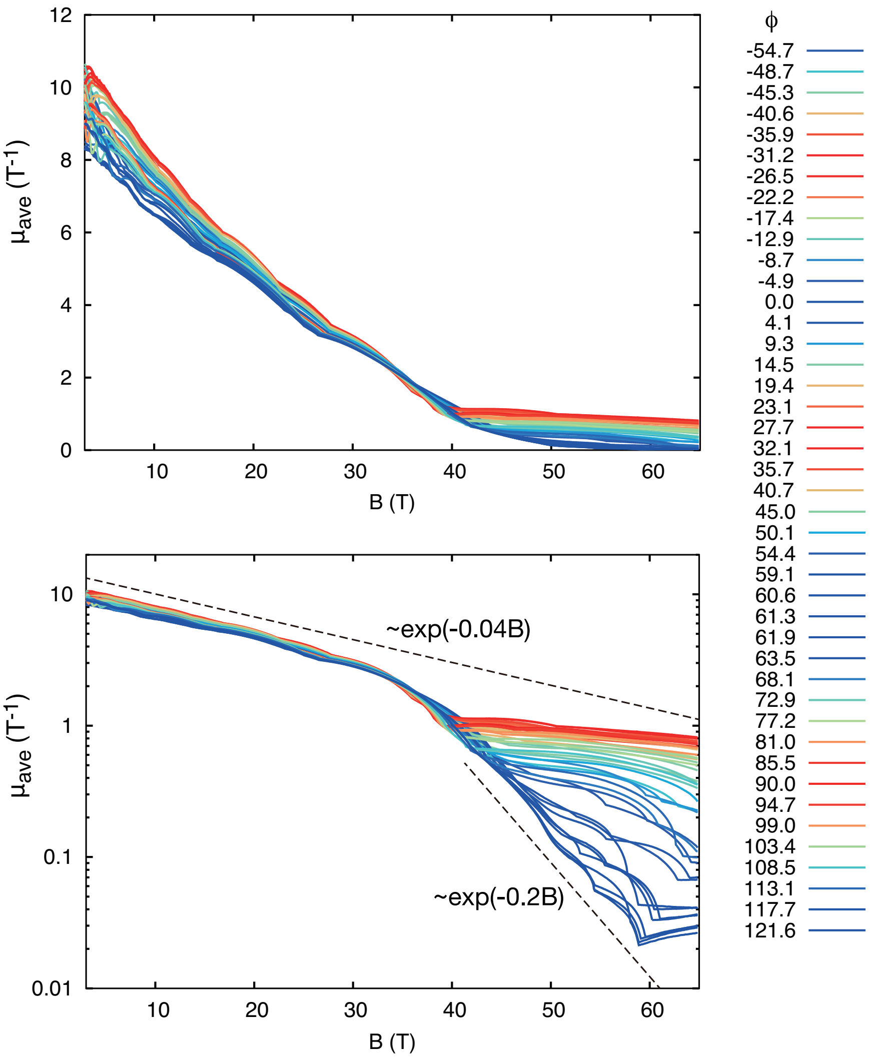

Supplementary Figure 2. **Field-dependence of averaged mobility** Averaged mobility $\mu_{\rm ave}$ as a function of magnetic field for different orientations in a linear (upper panel) and semi-logarithmic (lower panel) presentation. $\phi = 0$ corresponds to the bisectrix orientation.

Field-dependence of "averaged" mobility:

Combining our experimental $\rho_{33}(B)$ and theoretical $n_h(B)$ for two orientations, we can obtain the magnitude of "averaged" mobility using the relation:

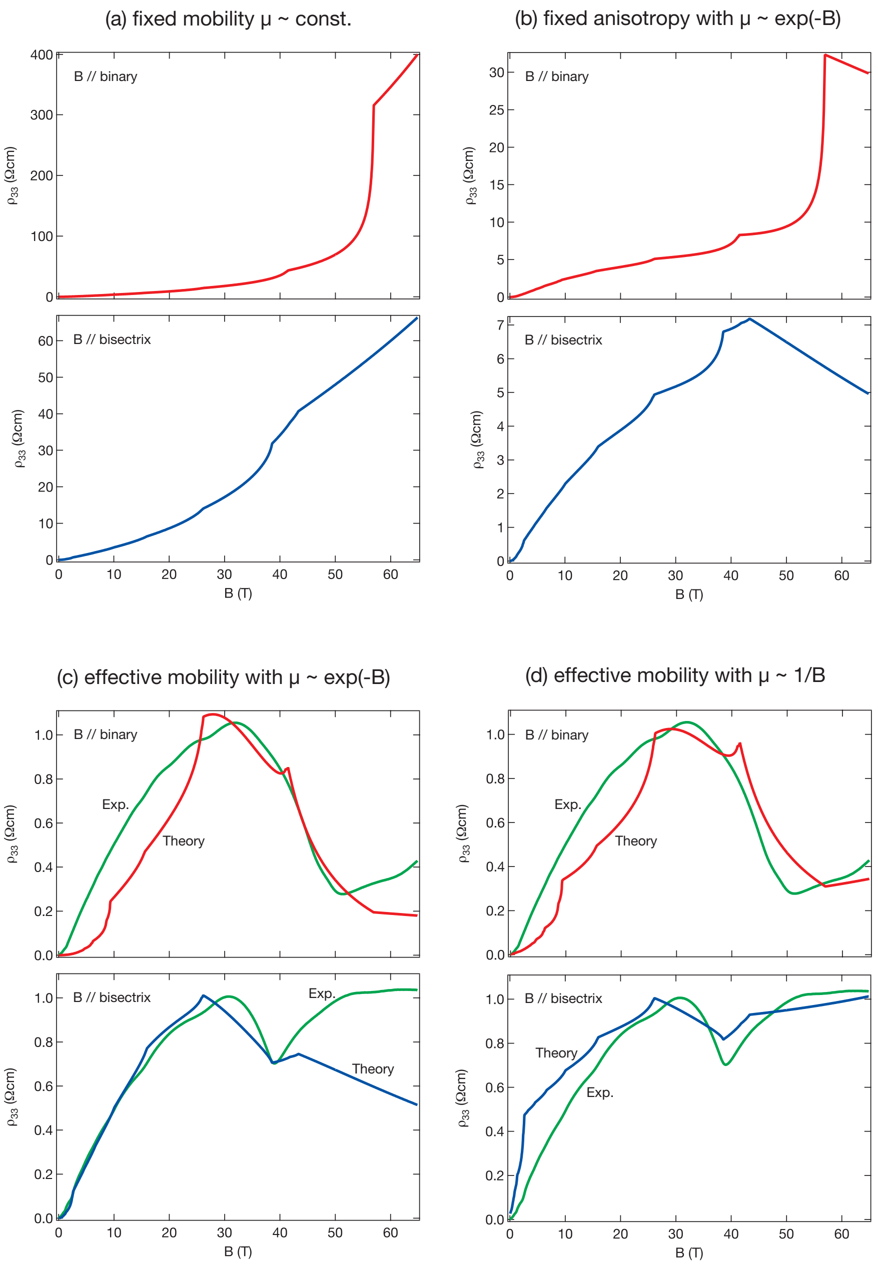

Supplementary Figure 3. **Four different scenarios** Magnetoresistivity with (a) fixed mobilities; (b) fixed anisotropy with field-dependence of $\mu \propto e^{-0.04B}$; (c) effective mobility with $\mu \propto e^{-0.04B}$; and (d) effective mobility with $\mu \propto 1/B$.

$$\rho_{33} = \frac{\mu_{\rm ave} B^2}{e n_h}. \tag{19}$$

Supplementary Fig. 2 shows the field-dependence of $\mu_{\rm ave}$ for each orientation of magnetic field. The field-dependence for both orientations follows $\mu_{\rm ave} \sim e^{-0.04B}$ below 30T and

much faster afterwards. The main reason for this change is that "averaged" mobility is a combination of different components of the mobility tensor and the relative weight of these components change as a valley begins to become empty. The open question is if this is the only reason for the anomalies seen around .

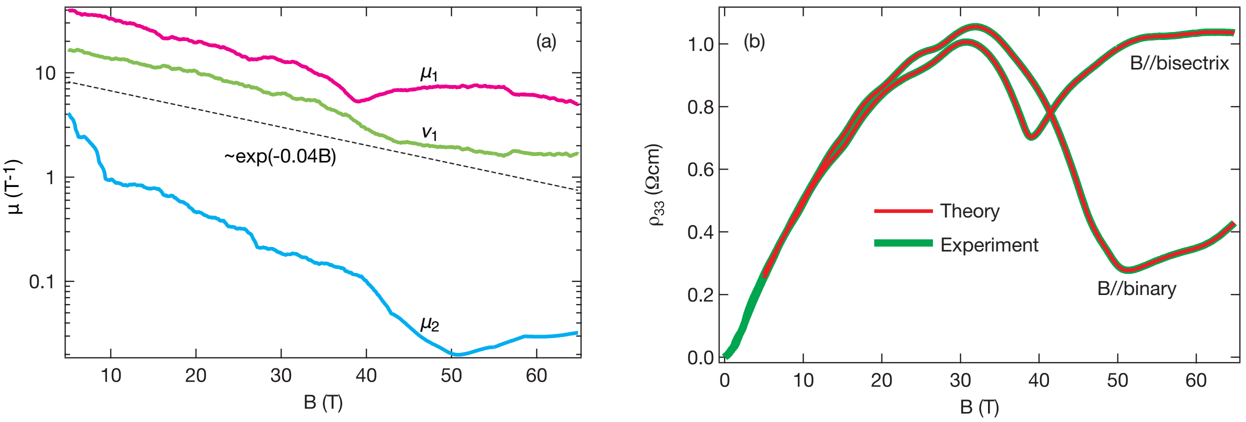

Supplementary Figure 4. **Non-monotonic field-dependence of mobility** (a) Field-dependence of μ_1 (red), μ_2 (blue) and ν_1 (green) in order to fit the experimental magnetoresistance through Supplementary Eqs. (4) and (5) with the theoretically obtained carrier density n_i. (b) Magnetoresistance so obtained theoretically (thin red lines) and experimentally (thick green lines).

Four different scenarios:

Taking this as a guide, we calculate the magnetoresistivity, using the theoretical n_a and n_b with three different assumptions on the field dependence of mobility: (a) keep the mobility constant and equal to its value at low field; (b) keep the anisotropy of the mobility equal to its value at low field, but assuming that its magnitude changes as $\mu \propto e^{-0.04B}$; (c) assuming that the field-dependence of mobility components follow $\mu \propto e^{-0.04B}$ with variable anisotropy; and (d) assuming that the mobility components follow $\mu \propto 1/B$ with variable anisotropy. Let us consider these four scenarios:

The first scenario is Fixed Mobility. Supplementary Fig. 3 (a) shows ρ_{33} as a function of B computed from Supplementary Eqs. (4) and (5) with constant mobilities $\mu_1 = 879$ T^{-1}, $\mu_2 = 47$ T^{-1}, $\nu_1 = 56$ T^{-1}, which are the values at 10 K and 0.5 T in Ref.[7]. (Note that the present sample has the RRR which is three to four times lower than that in Ref.[7]. Therefore, the mobility is also lower by such a factor. But the anisotropy should be very

similar.) As seen in Supplementary Fig. 3 (a), the magnitude of the magnetoresistivity is much larger than the experimental value of $\rho_{33} \sim 1$ Ωcm.

The second scenario is Fixed Anisotropy with $\mu \propto e^{-0.04B}$. It is reasonable to expect that the mobility is field dependent in general. Supplementary Fig. 3 (b) shows ρ_{33} with the mobility, whose amplitude changes as $\mu \propto e^{-0.04B}$ but anisotropy is kept from low fields as $\mu_1 = 879e^{-0.04B}$ T^{-1}, $\mu_2 = 47e^{-0.04B}$ T^{-1}, $\nu_1 = 56e^{-0.04B}$ T^{-1}. Although the magnitude is reduced, the theoretical ρ_{33} is still far from the experimental curve.

The second scenario Variable Anisotropy with $\mu \propto e^{-0.04B}$. Supplementary Fig. 3 (c) shows ρ_{33} with field-dependent effective mobilities as $\mu_a = 0.15e^{-0.04B}$ T^{-1}, $\mu_b = 1000e^{-0.04B}$ T^{-1}, $\mu'_a = 1000e^{-0.04B}$ T^{-1}, and $\mu'_b = 4.8e^{-0.04B}$ T^{-1}. There are clear drops at around B_{empt} both for $B \parallel$ binary and bisectrix, the amplitude of the drop for $B \parallel$ binary is larger than that for $B \parallel$ bisectrix, and the maximum of the magnetoresistivity is $\rho_{33} \sim 1$ Ωcm. These points roughly agrees with experiments. Above B_{empt}, however, the theoretical ρ_{33} keeps decreasing, while the experimental ρ_{33} increases. Presumably, this mismatch arises because μ above changes its field dependence above B_{empt} (See the behavior of μ_{ave} of Supplementary Fig. 2).

The fourth scenario is Variable Anisotropy with $\mu \propto 1/B$. We can assume that $\mu_a = 1.4/B$, $\mu_b = 1000/B$, $\mu'_a = 1000/B$, and $\mu'_b = 45/B$. (These functional forms are valid only at high fields, since it diverges at 0T.) In this case, it decreases slower than $\mu \propto e^{-0.04B}$ at high fields. As seen in Supplementary Fig. 3 (d), we find a better agreement between the theoretical and experimental ρ_{33}. However, the agreement between experiment and theory is still imperfect.

Non-monotonic field-dependence of mobility:

Fig. 4 (a), shows the best fit to the components of mobility tensors using the experimental magnetoresistivity and theoretical n_a and n_b and Supplementary Eqs. (4) and (5). As seen in Fig. 4 (b), there is a perfect agreement. Since there are three (μ_1, μ_2, and ν_1) variables and two equations, we cannot determine μ_1, μ_2, and ν_1 uniquely. As seen in Fig. 4 (a), all components of the mobility tensor show anomalies near 40 T.

In conclusion, the semiclassical picture combined with a phenomenological assumption on the field dependence of the mobility tensor leads to theoretical curves close to the experi-

mental one. However, we find that in all scenarios, an anomaly arises in the field-dependence of μ in the vicinity of B_{empt}. This anomaly in the field-dependence of mobility suggests that effects beyond the non-interacting picture of electrons and holes may play a partial role in the observed drop of magnetoresistance.

SUPPLEMENTARY REFERENCES

---

[1] Hiruma, K. and Miura, N. Magnetoresistance Study of Bi and BiSb Alloys in High Magnetic Fields. II. Landau Levels and Semimetal-Semiconductor Transition. J. Phys. Soc. Jpn. **52**, 2118-2127 (1983)

[2] Miura, N., Hiruma, K., Kido, G. and Chikazumi, S. Observation of the Magnetic-Field-Induced Semimetal-Semiconductor Transition in Bi under Megagauss Fields, Phys. Rev. Lett. **49**, 1339-1342 (1982)

[3] Zhu, Z., Fauqué, B., Malone, L., Antunes, A. B., Fuseya, Y. and Behnia, K., Landau spectrum and twin boundaries of bismuth in the extreme quantum limit, PNAS **109**, 14813-14818 (2012)

[4] Zhu, Z., Fauqué, B., Fuseya, Y. and Behnia, K. Angle-resolved Landau spectrum of electrons and holes in bismuth. Phys. Rev. B **84**, 115137 (2011)

[5] Vecchi, M. P., Pereira, J. R. and Dresselhaus, M. S., Anomalies in the magnetoreflection spectrum of bismuth in the low-quantum-number limit, Phys. Rev. B **14**, 298-317 (1976)

[6] Aubrey, J. E. Magnetoconductivity tensor for semimetals, J. Phys. F **1**, 493-497 (1971)

[7] Collaudin, A., Fauqué, B., Fuseya, Y., Kang, W. and Behnia, K. Angle Dependence of the Orbital Magnetoresistance in Bismuth. Physical Review X, **5**, 021022 (2015)
